# Can we breed hail-resilient wheat? Insights from a hail affected breeding trial

**DOI:** 10.3389/fpls.2026.1904031

**Published:** 2026-08-11

**Authors:** Arpit Gaur, Andrew Lehnerz, Duncan Pantos, Ricardo Leiton, Ronald Ramsfield, Suchismita Mondal

**Affiliations:** Department of Plant Sciences and Plant Pathology, Montana State University, Bozeman, MT, United States

**Keywords:** GWAS, hail resilience, hailstorm, spike, stem, wheat

## Abstract

Hail-induced yield loss threatens wheat-based food security across the globe, yet hail damage has largely remained unexplored as a quantifiable breeding phenotype. The present investigation provides insight from the variation observed in winter wheat breeding lines after a significant hail event in 2024. This investigation demonstrates that hail-induced mechanical damage and associated grain yield loss retain substantial heritable components with high entry-level (grain yield: 74.65%; hail damage score: 61.52%) and moderate plot-level heritability (grain yield: 59.30%; hail damage score: 43.49%). Grain yield exhibited moderate and significant negative correlation (r = -0.57) with hail damage score. Putative loci associated with hail response were identified across ten chromosomes of hexaploid wheat, delineated into seven quantitative trait loci. These quantitative trait loci largely co-localized with previously reported loci associated with stem strength, lodging, and spike architecture-related traits. Functional annotation further revealed enrichment of candidate genes belonging to functional categories with potential roles in stem and spike morphology, supporting a plausible biological basis for genetic variation in hail response in wheat. Together, these findings suggest hail damage as a genetically tractable polygenic trait, providing a foundation for incorporating physical resilience directly into wheat improvement programs to enhance resilience in wheat production system.

## Introduction

Hailstorms are destructive, spatially heterogeneous, and unpredictable weather events, causing over USD 1 billion in annual agricultural losses worldwide (Battaglioli et al., 2025). These events frequently affect major wheat-growing regions, including the Northern Great Plains of the United States, Indo-Gangetic Plains, China, parts of Argentina and Africa, and Australia. Collectively, these regions account for approximately 90% of global wheat production (Erenstein et al., 2022). Given that wheat is a staple crop, with per capita consumption exceeding 50 kg annually, hail events represent a direct threat to wheat production and global food security. Despite their economic and agronomic importance, hail-induced damage remains largely unexplored as a quantifiable breeding phenotype, largely because stochastic mechanical damage is difficult to standardize and reproduce experimentally and natural hail events are inherently unpredictable, limiting systematic phenotyping experiments.

Many studies advocate that hail-induced crop losses are largely determined by hailstorm intensity, hailstone size, crop type, crop growth and canopy position (Holman et al., 2023; Sánchez et al., 1996). Few studies have demonstrated post-hail management strategies to promote plant recovery (Singh Dhillon et al., 2021). However, post-hail management may not be feasible in large field and cereal crops like wheat. Varietal differences in response to hail damage may reflect heritable variation in plant architecture, stem strength, leaf angle, canopy density, developmental timing, and capacity for post-injury recovery. These traits could influence both the initial severity of mechanical injury and the ability of plants to maintain reproductive potential after damage.

Reitz (1942) observed non-random variations among winter wheat hybrids and pure lines for stem breakage (9% to 83%) and shattering (2% to 28%), while hypothesizing straw height and stiffness as possible function of resilience. Likewise, a post-hail observation study in potato crop demonstrated that crop cover positively correlates (*R^2^* = 0.74) with grain yield, while varieties with bigger stem diameter shows lower stem breakage that negatively correlates (R^2^ = 0.80) with tuber yield (Jin et al., 2014). Therefore, although hail events are environmentally stochastic, the observed plant response may still contain a genetic component that is relevant for selection. Quantifying these variations under naturally occurring hail damage provides a rare opportunity to evaluate whether hail resilience can be treated as a measurable trait rather than an incidental field observation.

A review of the literature reveals a long-standing gap in the systematic evaluations of genetic variation in varietal response with respect to hail damage in wheat. To date, Neatby (1940) and Reitz (1942) remain the only studies providing systematic evidence for variation for hail resistance in wheat. Within this context, to our knowledge, this study presents the first genome-wide association analysis (GWAS) aimed at dissecting the genetic architecture of natural hail resilience in wheat, bridging an 80-year gap in the literature. We leveraged a natural hail event that impacted the Montana State University winter wheat breeding program (Bozeman, USA) in August 2024, during which substantial variation in mechanical damage was observed among breeding lines. This natural perturbation enabled us to address a fundamental question: Can hail damage be quantified as a genetically influenced and selectable trait in wheat breeding?

## Materials and methods

### Material

The population studied comprised of 320 winter wheat breeding lines representing six advanced breeding nurseries (**Table 1**). Each nursery was planted on October 15, 2023, in a lattice design at Arthur H. Post Agronomy Farm, Bozeman, USA. The crop was harvested on August 10 and 11, 2024. However, the number of replications varied between trials (**Table 1**). Crop management followed the standard package-of-practices recommended by Montana Agricultural Extension Services.

**Table 1:** List of advanced winter wheat breeding nurseries of Montana State University included in the present investigation.

Sr. no.	Nursery	Acronym	Population Size*	Replication
1.	Advanced Yield Trial	AYT	36	3
2.	Forage Observation Nursery	FO	119	2
3.	Preliminary Yield Trial-A	PYA	64	2
4.	Preliminary Yield Trial-C	PYC	36	2
5.	Preliminary Yield Trial-D	PYD	45	2
6.	Sawfly Yield Trial	SF	49	2

*Population size includes repeated checks across trials.

### Hail event and scoring

On the afternoon of August 6, 2024, two to three high-intensity hail events occurred between 16:00 and 16:30 pm across Gallatin County, including the Montana State University Winter Wheat Breeding site at the Arthur H. Post Agronomy Farm (45°67′ N, 111°15′ W). Each hail event lasted approximately 3–5 minutes.

On the following day, plot level hail damage (HLD, %) was assessed on a 0–100% scale, where 0% represented no visible mechanical damage and 100% represented complete plant loss. In this investigation to assess the mechanical damage overall defoliation, stem breakage, spike breakage, and grain shattering were considered. A representative of 10% plot damage and 80% of plot damage, which was used as a reference has been provided in **Supplementary Figure 3**.

### Statistical analysis

A generalized additive model (Wood, 2025) was independently fitted for each nursery using “mgcv” package in R (Wood, 2000) to analyze the hail damage (HLD: %) and grain yield (GY: g m^-2^), while accounting for spatial heterogeneity and experimental design effects. The model can be mathematically expressed as: $y_{ijkl}=\mu +f\left(\;row_{i}\times column_{j}\right)+replication_{k}+genotype_{l}+\epsilon_{ijkl}$. Where, *μ* is overall mean and *f*(*row_i_*×*column_j_*) is a two-dimensional tensor-product smooth accounting for both large-scale and local spatial heterogeneity arising from uneven hail distribution across the field. Sequential Type-I analysis of variance (ANOVA) was performed to estimate the contribution of each component toward the phenotype. Heritability was estimated at plot- $\left(h_{p}^{2}=\frac{\sigma_{g}^{2}}{\sigma_{g}^{2}+\sigma_{e}^{2}}\;\right)$ and entry-levels $\left(h_{e}^{2}=\frac{\sigma_{g}^{2}}{\sigma_{g}^{2}+{\frac{\sigma_{e}}{r}}^{2}}\;\right)$ to measure the proportion of variation due to genetic effect. Where, $\sigma_{g}^{2}$ and $\sigma_{e}^{2}$ are genotypic and error variances while *r* is number of replications.

Best linear unbiased estimates (BLUEs) for each genotype were extracted using the “emmeans” package in R (Lenth and Piaskowski, 2017) to obtain adjusted genotype means after accounting for spatial and experimental design effects.

### Genome-wide association study

Winter wheat forage breeding lines (*n* = 119) were eliminated prior to any genomic analysis, given the strong population influence. Henceforth, analyses are based on 211 genotypes. Genotyping-by-sequencing was performed at the Central Small Grains Genotyping Laboratory, USDA-ARS, Manhattan, Kansas, and an unfiltered processed data comprising genome-wide single nucleotide (SNP) marker information on a total of 211,255 positions based on IWGSC RefSeq v2.1 (Zhu et al., 2021) was received. Data filtering was then performed in TASSEL (Bradbury et al., 2007) to retain most informative SNPs and genotypes using the following thresholds: minor allele frequency (MAF) ≥ 0.05, site call rate ≥ 90%, genotype call rate ≥ 90%, and heterozygosity ≤ 10%. Filtering resulted in 16,039 informative SNPs and 189 genotypes for subsequent analyses. The population structure was inferred using STRUCTURE software v2.3.4 (Pritchard et al., 2000) for K = 1 to 10 with 10 independent runs per K. The optimal number of subpopulations was determined using the Δ*K* method (Evanno et al., 2005) as implemented in STRUCTURE HARVESTER (Earl and vonHoldt, 2011). A compressed mixed linear model GWAS was performed in GAPIT-R (Lipka et al., 2012) to identify putative loci at cutoff *-log10(p)* ≥ 3.0, where first 3 principal components along with K-matrix were used to minimize the effect of population structure. Genome-wide linkage disequilibrium (LD) decay was estimated by fitting a LOESS curve, using “ggplot2” package in R (Wickham, 2016), between pairwise LD (*r*^2^) values and physical distance between SNPs as calculated using TASSEL. The estimated LD decay distance was subsequently used to delimit adjacent significant SNPs into putative QTL regions. It should be noted that in the present investigation phenotypic variance explained (PVE, %) has been calculated using following formula:


$$PVE=\frac{2.\;\beta^{2}.MAF.\left(1-MAF\right)}{2.\;\beta^{2}.MAF.\left(1-MAF\right)+SE^{2}.2.N.MAF.\left(1-MAF\right)}\times 100$$

Where, *β* is effect size, MAF is minor allele frequency, SE is standard error of *β*, and N is sample size.

### Putative candidate genes

High confidence IWGSC RefSeq v2.1 genes were retrieved within the ±1 Mbp window of each putative SNP, along with genomic coordinates, and associated InterPro and gene ontology terms and descriptions. A BLAST-based functional annotation was also performed in funPlantGenes tools (Cao et al., 2026) by employing SeqToFunc module, which allows to predict functions of genes of interest based on gene similarity against functionally curated genes. We then identified biologically relevant genes based on functional annotations and extensive literature review. REVIGO (Supek et al., 2011) was additionally employed to summarize and biologically contextualize enriched gene ontology terms. *In silico* expression profiling of putative candidate genes was performed using WheatOmics (Ma et al., 2021) after converting IWGSC RefSeq v2.1 stable gene IDs to corresponding IWGSC RefSeq v1.1 stable gene IDs.

## Results

### Hail damage showed heritable genetic variation

**Figure 1:**
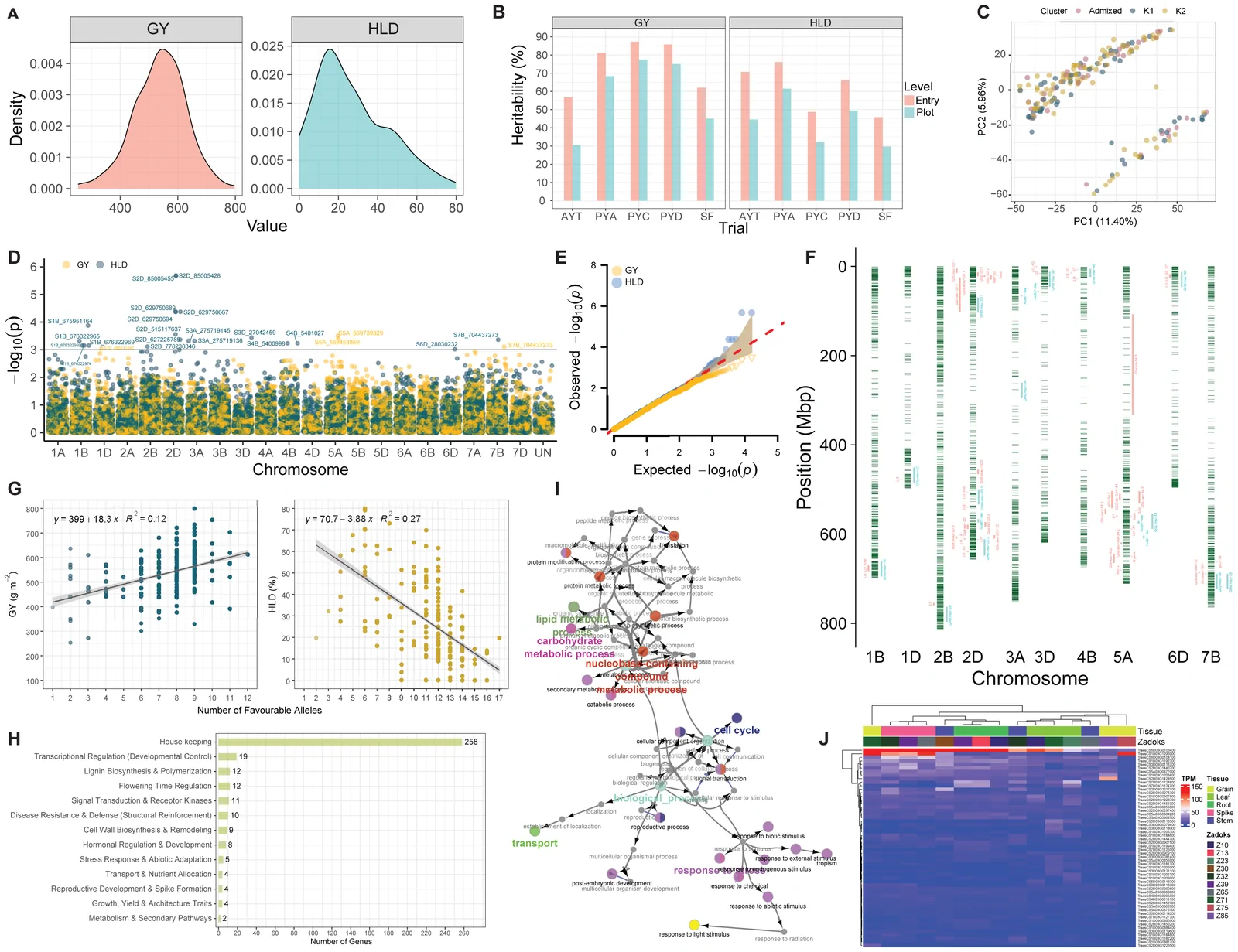
Genetic architecture and biological basis of hail-resilience in winter wheat. **(A)** Density distribution of hail-induced grain yield loss (GY, g m^−2^) and mechanical hail damage (HLD, %) observed across winter wheat breeding lines, demonstrating substantial phenotypic variation suitable for the present genomic investigation. **(B)** Entry- and plot-level heritability estimates for hail-induced GY and HLD across six advanced winter wheat breeding trials of the Montana State University Winter Wheat Breeding Program, indicating substantial heritable genetic components underlying both traits. **(C)** Principal component analysis (PCA) biplot revealing two major genetic clusters with several admixed individuals. The first two principal components explained 17.36% of the total genetic variation. **(D)** Manhattan plot showing the distribution of −log10(p) values for single nucleotide polymorphism (SNP) markers associated with GY (golden) and HLD (blue) across the 21 wheat chromosomes and unmapped scaffolds. **(E)** Quantile–quantile (Q–Q) plot illustrating the agreement between observed and expected GWAS signals for GY and HLD at −log10(p). **(F)** Linkage map showing the chromosomal positions of quantitative trait loci (QTLs) identified in the present investigation (blue), delineated using the genome-wide linkage disequilibrium (LD) decay threshold of 15.4 Mbp, alongside previously reported QTLs and genes (red) associated with stem strength and spike morphology. **(G)** Linear regression between the number of favorable alleles and observed GY and HLD, indicating the quantitative nature of hail resilience and the potential utility of marker-assisted selection for hail-resilient wheat breeding. **(H)** Distribution of 371 putative candidate genes identified within a ±1 Mbp interval surrounding significant SNPs, classified into 13 functional categories based on their putative biological roles. **(I)** Gene ontology-based biological process network of putative candidate genes, revealing that the majority of genes are associated with plant architecture, metabolic regulation, stress response, and developmental processes. **(J)** Spatiotemporal expression profiling of 56 biologically relevant putative candidate genes associated with hail resilience across multiple tissues and developmental stages in wheat.

To account for the spatial heterogeneity in hail impact, we generated a spatial hail damage map, which revealed strong localized variation in hail injury across rows and columns, confirming that the hail event imposed heterogeneous mechanical stress across the field (**Supplementary Figure 1**). To adjust for the spatial effect, we fitted a spline model within each trial. We observed broad, continuous phenotypic variation in adjusted genotypic means for both grain yield (GY, g m^-2^) and hail damage (HLD, %) (**Figure 1A**). Overall, GY ranged from 253.28 g m^-2^ to 797.73 g m^-2^, while HLD ranged from 0% to 73.82% (**Supplementary Table 1**). Trials showed substantial differences in population mean of both GY and HLD. The lowest and highest population means for GY were observed in the preliminary advanced yield trial (PYA: 510.56 g m^-2^) and advanced yield trials (AYT: 567.35 g m^-2^), respectively. Likewise, sawfly trial (SF) showed the lowest average hail injury (9.66%) whereas, PYA showed the highest average hail injury (36.08%).

Analysis of variance from the *generalized additive model* revealed significant genotypic effects across trials, whereas effect of replication was generally nonsignificant, and effect of row and column were generally significant (**Supplementary Table 2**). These results indicated that variation in GY and mechanical damage was primarily genotype specific.

Broad-sense heritability was estimated at both entry $\left(h_{e}^{2}\right)$ and plot $\left(h_{p}^{2}\right)$ levels, and both varied across trials (**Figure 1B**). Among trials, the minimum $h_{p}^{2}$ for GY and HLD were observed for AYT and SF trials, and the maximum $h_{p}^{2}$ were reported for PYA and PYC (preliminary yield trial C), respectively. Similarly, at entry level, AYT and SF showed the lowest heritability for GY and HLD, respectively, whereas PYA and PYC showed the highest heritability for GY and HLD, respectively. Overall, high $h_{e}^{2}$ values (GY: 74.65%; HLD: 61.52%) and moderate $h_{p}^{2}$ values (GY: 59.30%; HLD: 43.49%) indicated that hail injury and post-hail event GY recovery were genetically tractable. Furthermore, a moderate but significant negative association (*r* = -0.57, *p ≤ 0.05*) was reported between GY and HLD across trials. The significant negative correlation was also consistent within PYA (r = -0.78, *p ≤ 0.001*), PYC (r = -0.65, *p ≤ 0.001*), PYD (r = -0.53, *p ≤ 0.001*), and SF (r = -0.47, *p ≤ 0.001*). Interestingly, for AYT non-significant positive correlation (r = 0.13, *p=0.44*) was reported.

### Genetic loci associated with hail damage and favourable allele accumulation

We retained a total of 16,039 informative SNPs following stringent quality control, which were used in the subsequent analyses. The lowest (156) and highest (1,667) numbers of informative SNPs were identified on chromosomes 4D and 2B, respectively. In addition, a total of 175 SNPs carried multiple anchoring positions and were treated as unmapped marker. The highest number of informative SNPs was identified on subgenome B (7,025), followed by subgenomes A (5,637) and D (3,202).

We further performed population structure analysis to assess genetic diversity and select the appropriate GWAS algorithm. Evanno’s method for identifying the optimal population structure revealed two peaks of Δ*K* at *K* = 2 (~750) and *K* = 4 (~600). However, the corresponding principal component analysis of genetic divergence, using all informative SNPs, confirmed that the panel exhibited two subpopulations (*K* = 2) with admixture (**Figure 1C**; **Supplementary Figures 2A, B**; **Supplementary Table 3**). Of the 189 genotypes, 60 represented subpopulation K1 and 91 represented subpopulation K2, whereas the remaining 38 carried highly shared ancestry from both subpopulations.

Given the structured population, we used compressed mixed linear model for GWAS, resulting in a total of 23 significant SNPs (*-log_10_(p)* ≥ 3.0) across the traits. Significant associations were identified on 10 of the 21 chromosomes (1B, 1D, 2B, 2D, 3A, 3D, 4B, 5A, 6D, and 7B; **Figure 1D**). Maximum (7) significant SNPs were detected on chromosome 2D, followed by chromosome 1B (5). For GY and HLD, three and nineteen independent SNPs were identified, respectively, along with one pleiotropic SNP, S7B_704437273 (**Supplementary Table 4**). These loci explained 5.82-6.68% of the phenotypic variance (PVE) for GY and 5.63-11.27% for HLD, supporting the quantitative genetic basis of hail resilience. We did not observe any early deviation in the quantile-quantile plots (**Figure 1E**) for either of the traits, which indicated the statistical robustness of the present GWAS and supported true association at the selected significance threshold.

LD decay analysis was performed to delineate linked SNPs and define QTL intervals. Genome-wide LD decayed to the selected cutoff of *R²* = 0.10 at approximately 15.4 Mbp (**Supplementary Figure 2C**). Subgenome A showed a sharper decline comparable to the genome-wide trend, whereas subgenomes B and D retained higher LD over longer physical distances.

Significant SNPs were delineated into seven QTLs using genome-wide LD decay (**Figure 1F**). The maximum number of significant SNPs (5) were reported within QTL *QHld.msu-1B* (1B: 660,551,164:694,722,974 bp), which cumulatively explained 33.7% of phenotypic variation. The strongest QTL for GY was identified on chromosome 5A (*QGy.msu-5A*) between 554,053,869 and 588,139,329 bp, with two SNPs cumulatively explaining 13.4% phenotypic variation.

Allelic effect analysis showed that alternative alleles at these SNPs shifted trait distributions for both GY and HLD (**Supplementary Figure 4**). Because the dataset was imbalanced, statistical significance testing was not feasible. Nonetheless, favorable alleles demonstrated cumulative directional trend (**Figure 1G**), with increased GY (*r* = 0.35) and reduced HLD (*r* = -0.52), indicating the potential of trait improvement through marker-assisted selection.

### Putative candidate genes

Within ±1 Mbp windows of significant SNPs, we identified 1,602 high-confidence genes (HCGs) from the IWGSC RefSeq v.2.1 assembly, including seven genes that directly overlapped significant SNPs. Functional annotation integrating Gene Ontology (GO), InterPro domains, and BLAST-based homology using PlantEnsembl database and funPlantGenes tool yielded putative functions for 371 HCGs. Of these, 258 HCGs were classified as housekeeping genes, while 113 fell into 13 other functional categories with potential roles in stem and spike morphology (**Figure 1G**; **Supplementary Tables 5**–**8**). We identified a total of 262 high confidence GO terms associated with these HCGs (**Supplementary Table 5**). These terms represented 110 biological processes, 110 molecular functions, and 42 cellular components. The biological network (**Figure 1I**), developed using explained biological processes, showed high relevance to hail resilience. The *in-silico* gene expression profiling showed that 56 of 252 genes had expression levels ≥0.5 TPM in at least one wheat tissue or developmental stage (**Figure 1J**; **Supplementary Table 10**). Collectively, these analyses identified biologically relevant candidate genes across all hail-associated QTLs.

## Discussion

Our study demonstrates that mechanical injuries and subsequent grain yield losses due to hail events are not merely functions of random field exposure but also contain measurable and potentially selectable genetic components. Although the spatial analysis of hail events across the MTWW field indicated substantial spatial heterogeneity, the spatially adjusted values showed wide variation for the GY and HLD. Significant genotypic effects together with moderate to high plot- and entry-level broad sense heritability observed for both hail damage and post-hail GY, indicate differential and heritable varietal response to this natural perturbation. Relatively high entry-level heritability for both traits suggests that breeding for improved hail tolerance is feasible (Schmidt et al., 2019). In contrast, moderate plot-level heritability suggests that plot-level observations were influenced by spatial distribution of hailstones. Therefore, reliable selection for hail resilience will require spatial adjustment and multi-environment evaluations.

In this study, we reported a significant negative correlation between GY and HLD, which supports agronomic relevance of our scoring. A negative correlation between yield and hail-induced injuries is expected. However, this association was only moderate, which highlights that GY did not always decrease with increasing HLD scores, suggesting variable varietal responses; *i.e.*, specific genotypes may have either avoided mechanical injury or maintained superior GY despite mechanical injury. Although, five of the six breeding populations exhibited strong negative and significant correlation between GY and HLD, the AYT population showed a weak positive and non-significant correlation. This result, therefore, should not be interpreted as evidence that GY increase with hail damage, the observed association likely reflects possible population specific confounding effects which may include yield potential of genotypes and structural traits.

Hail-induced yield losses can result from defoliation, stem breakage, spike breakage, and grain shattering (Singh Dhillon et al., 2021). In this study, we observed the lowest population mean for hail damage with SF trial and the highest with PYA. This contrast may be partially explained by differences in stem architecture. Our SF and PYA trials comprised stable solid/semi-solid stem and hollow stem genotypes, respectively. Presumably greater pith density and lignin content in solid/semi-solid stem genotypes may have provided greater mechanical resistance to hail-induced lodging, breakage, or tissue injury. The AYT trial further showed wide variation in HLD, with overall moderate injury and the highest grain yield, because it comprised diverse plant architectures and generally high-yielding lines advanced from preliminary trials (PYA, PYC, PYD, and SF). Notably, our breeding population also carries substantial variation in spike structure (compact and loose types). We observed that compact spike types appeared to show reduced hail-induced shattering (**Supplementary Figures 3A–C**) in contrast to the loose spike type (**Supplementary Figures 3D–F**).

Our findings are consistent with Reitz (1942), who reported that yield loss in winter wheat genotypes with stiffer and/or shorter stem exhibited lower yield loss under natural hail damage. Reitz (1942) also observed that genotypes classified as “difficult to thresh” had less beaten grain from the spike, whereas variety Kawvale, a variety with natural tendency of shattering, showed substantial shattering loss due to hail impact. Similarly, Kolech et al. (2017) and Jin et al. (2014) reported that genotypes with thick stems and strong leaves sustain hail damage and show less yield loss in potato. Overall, these studies strengthen our findings and support the hypothesis that stem and spike architectural traits can provide hail resilience.

This study exemplifies an opportunistic investigation leveraging a natural perturbation. Therefore, systematic data to directly support above proposed hail-resilient mechanism was unavailable. Therefore, we performed GWAS, hypothesizing that if variation in HLD and GY was associated with stem and spike architecture, significant SNPs would coincide with or be anchored near existing loci for architectural traits.

Given that the present GWAS panel exhibited a structured population, we chose the CMLM (Pritchard et al., 2000), which resulted in a population-structure corrected GWAS and yielded a total of 23 significant SNPs, of which one was pleiotropic for GY and HLD. The pleiotropic locus suggested genetic association between these traits. We surveyed the literature to identify previously known QTLs, SNPs, and genes associated with stem and spike architecture. Additionally, we searched for orthologs and homologs of domestication genes *Btr1* (*Brittle Rachis 1*) and *Q*, which regulate rachis fragility (grain shattering) and free threshability (Vavilova et al., 2020), using pBLAST (**Supplementary Table 9**).

As expected, all the significant SNPs were located on chromosomes with known importance for architectural traits, while most of the SNPs co-localized with previously reported QTLs (**Figure 1F**; **Supplementary Table 10**) associated with stem and spike architectural traits such as stem elasticity, lodging tolerance, spike density, and compactness (Sun et al., 2017; Wang et al., 2025). Several QTLs also co-localized with or were proximal to *Vrn* and *Rht* genes. Notably, *QHld.msu-3D* and *QGy.msu-5A* overlapped with *HvBtr1*-like genes. *QGy.msu-5A*, located on the distal end of the long arm of chromosome 5A, was anchored within a known spike morphology hotspot affecting threshability and grain shattering, alongside domestication genes *Btr1* and *Q*. These results strengthen the evidence that variation in GY and HLD was influenced by stem and spike morphology.

Candidate gene analysis provided additional biological support for these mechanisms. Genes located within ±1Mbp windows (Gaur et al., 2022) of significant loci were enriched for functions related to transcriptional regulation, lignin biosynthesis, cell wall remodeling, receptor kinase signaling, stress response, and reproductive development. For instance, we identified genes containing glycosyltransferase domains, pectin methyltransferase domains, R2R3‐MYB transcription factors, and auxin response factors, which affect stem strength or elasticity through structural remodeling (Dai et al., 2026; Mitchell et al., 2007; Rocchi et al., 2012; Tang et al., 2023). Likewise, we identified several transporters, flowering regulators and WRKY-domain genes with reported role in spike architecture and spike-weight determination (Lin et al., 2024; Zhu et al., 2016). Additionally, the GO biological pathway network was consistent with these findings, supporting our hypothesis for hail-resilience mechanism in the present GWAS panel. Expression evidence in stem and spike tissues further supported the relevance of these candidates, although functional validation will be required to determine their direct roles in hail resilience.

In conclusion, this study provides a preliminary framework and serves as proof-of-concept for how we approach a long-standing threat to global agriculture. While hailstorms have traditionally been treated as unpredictable environmental shocks that significantly lead to wheat yield losses and hampers regional socioeconomic growth, our findings demonstrate that hail-induced mechanical injuries and grain losses are not solely determined by field exposure and may possess quantifiable and potentially selectable components. The key limitation of this study is that findings are based on a single growth stage and natural hail event. Therefore, the identified loci and candidate genes must be interpreted as preliminary genomic signals requiring validation across additional environments, populations, and controlled mechanical stress assays. Nonetheless, capturing a natural hail event within an active breeding program provided a rare opportunity to evaluate real-world mechanical damage under real agronomic conditions. Together, these findings suggest hail damage as a genetically tractable polygenic trait, providing a foundation for incorporating physical resilience directly into wheat improvement programs to enhance resilience in wheat production system. However, reliable selection for hail resilience will require spatial adjustment and multi-environment evaluations. By leveraging spatial hail patterns and multi-environment testing, breeding programs can more effectively characterize resilience traits, transforming environmental variability into actionable information for cultivar improvement.

## Data Availability

Data is available in the local MSU repository at https://www.lib.montana.edu/services/data/repositories/.
