# Supplementary material for: Can we breed hail-resilient wheat? Insights from a hail affected breeding trial
Source: Front Plant Sci. 2026 Aug 11;17:1904031. doi: 10.3389/fpls.2026.1904031 (PMC13503530; doi:10.3389/fpls.2026.1904031)

Figure S1. Spatial distribution of hail damage accessed on a scale of 0-100%.

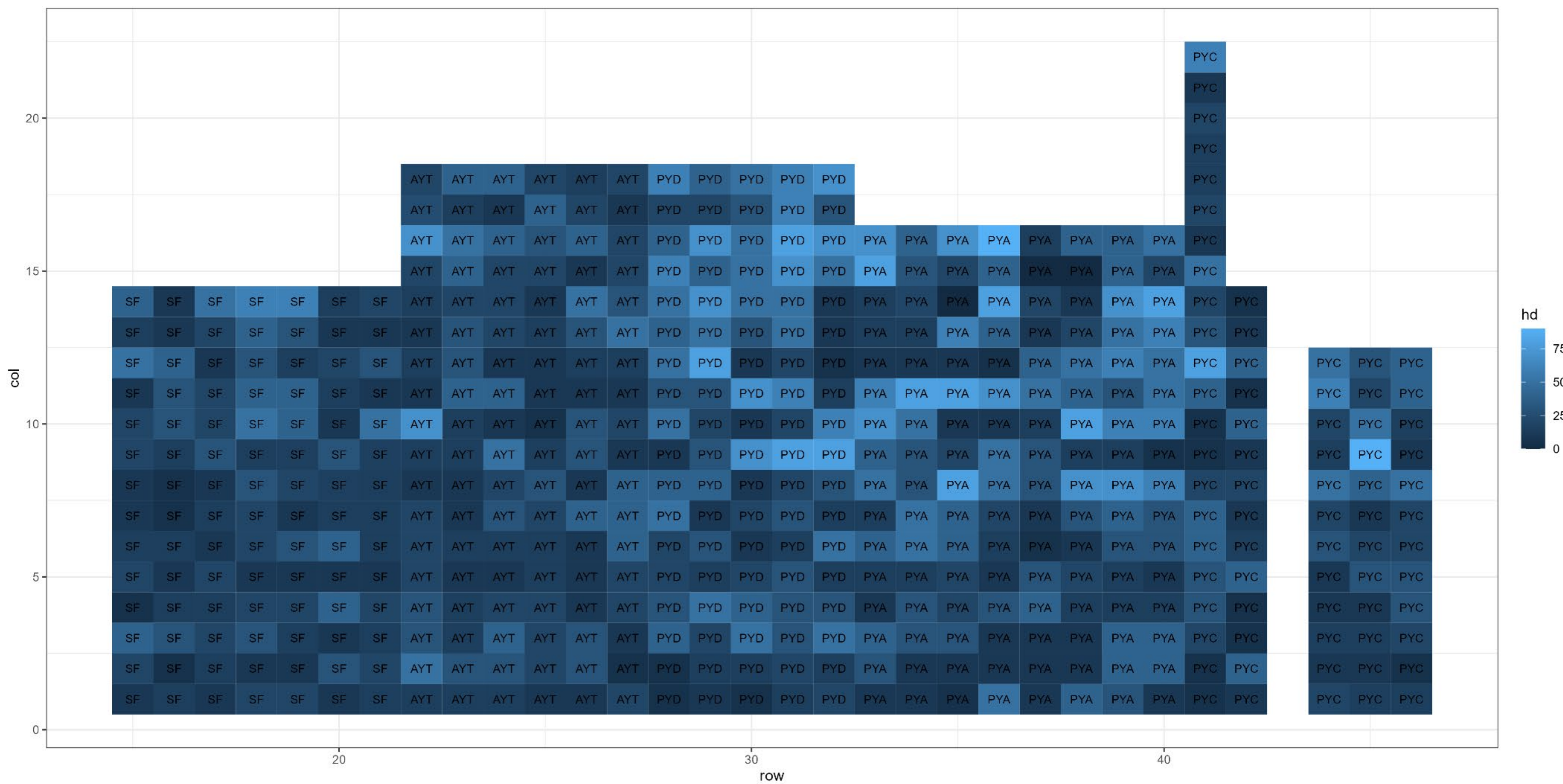

Figure S2. Population structure and linkage disequilibrium analysis of the GWAS panel. (A) ΔK values across different assumed population sizes (K). B) Population structure showing two major subgroups inferred at the optimal K value. (C) Genome-wide linkage disequilibrium decay across the A, B, and D genomes

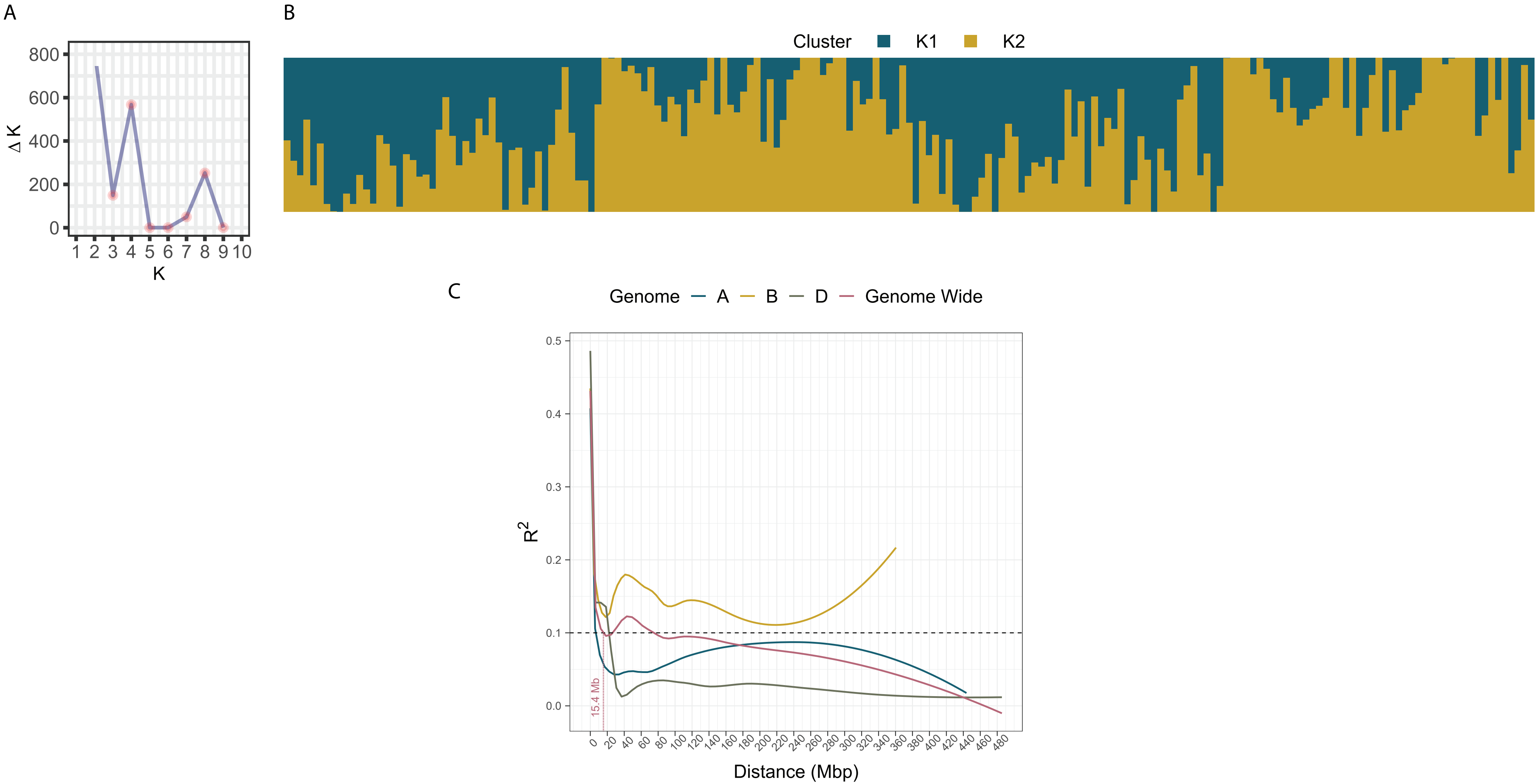

Figure S3. Photographs of the Montana State University winter wheat field (Bozeman, USA, 2024) captured two days after a hail event. The photographs show a winter wheat genotype with compact spikes exhibiting minimal spike breakage (A-B) and reduced shattering loss on the ground (C). In contrast, a genotype with loose spike morphology exhibited severe spike breakage (D-E) and greater shattering loss on the ground (F).

A

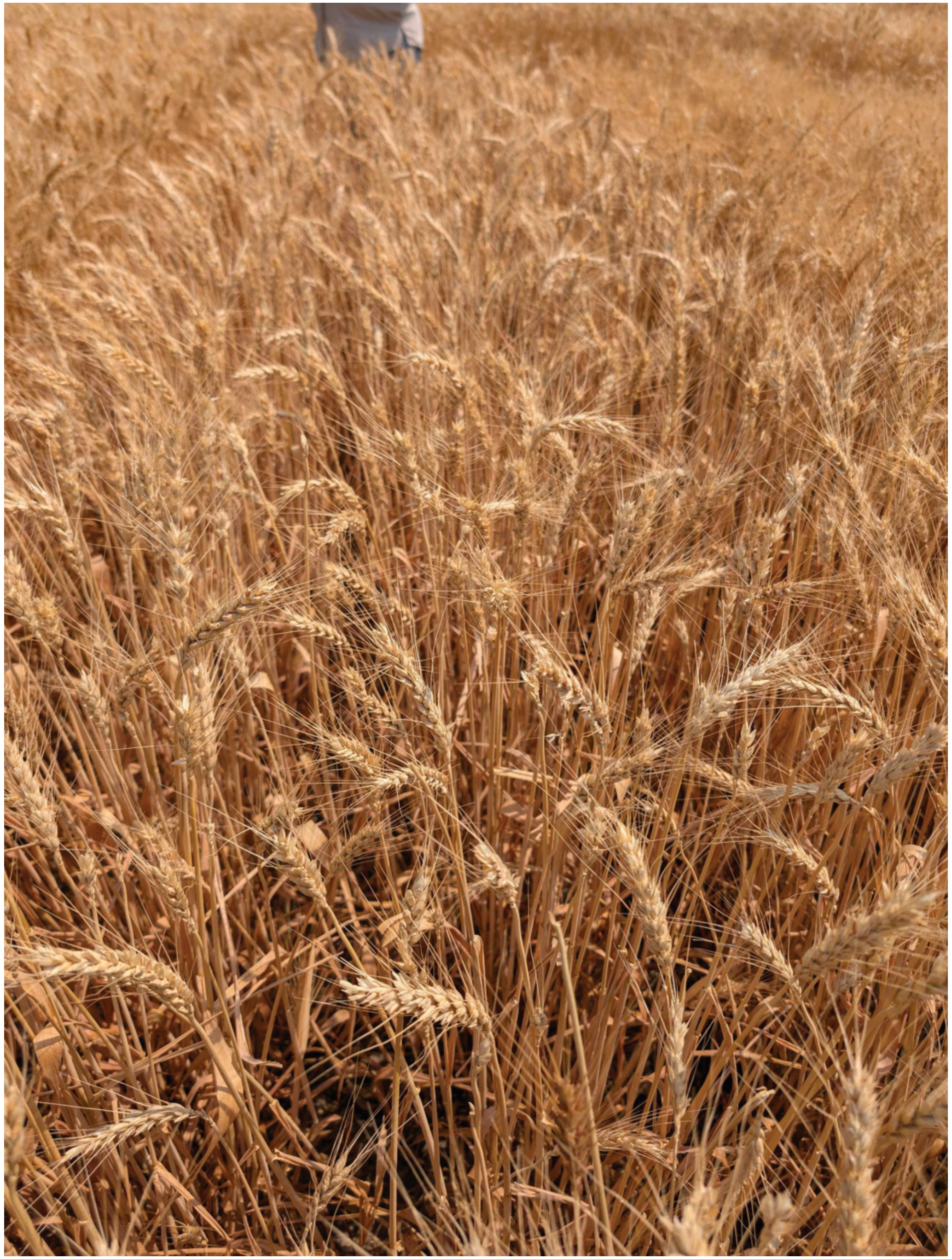

B

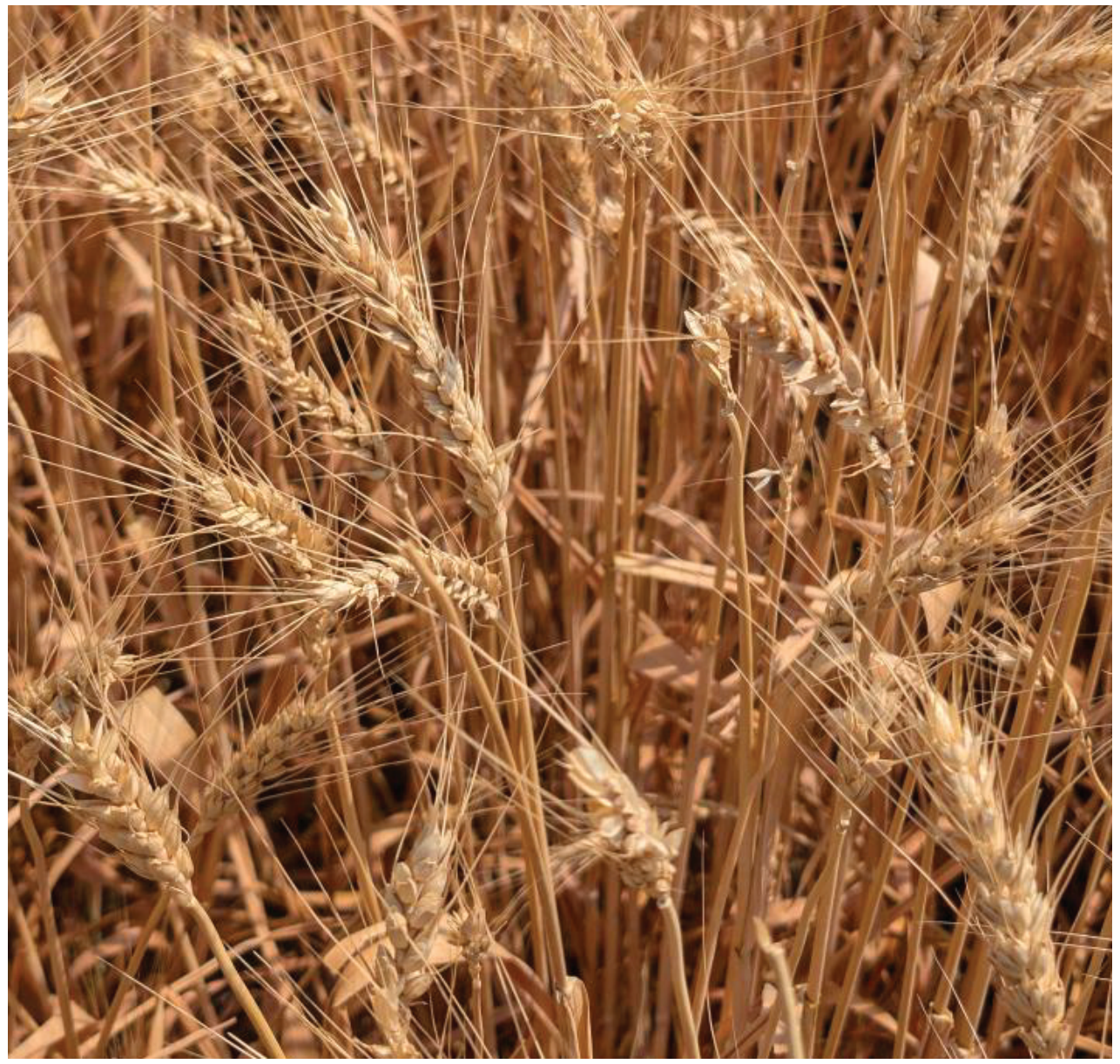

C

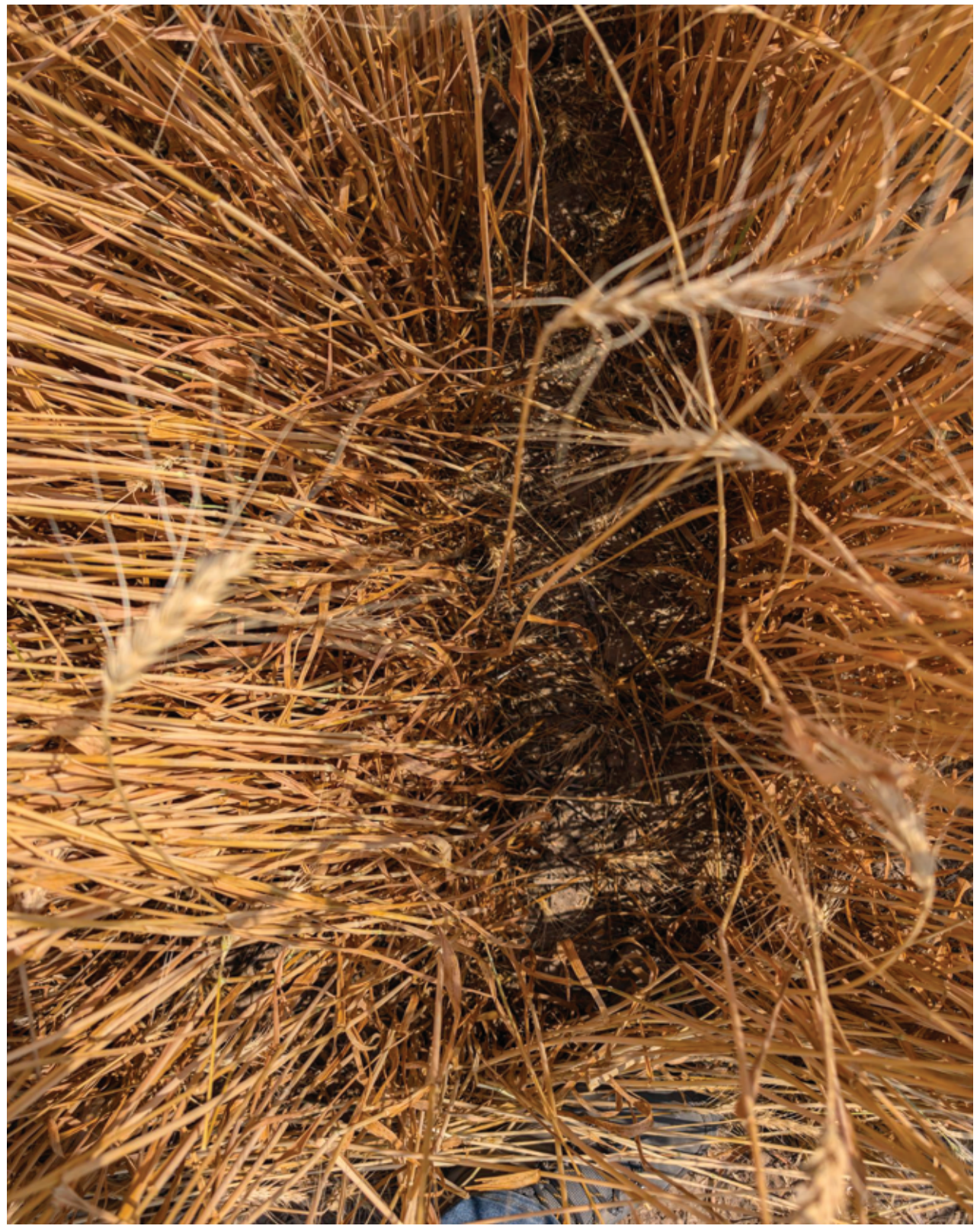

D

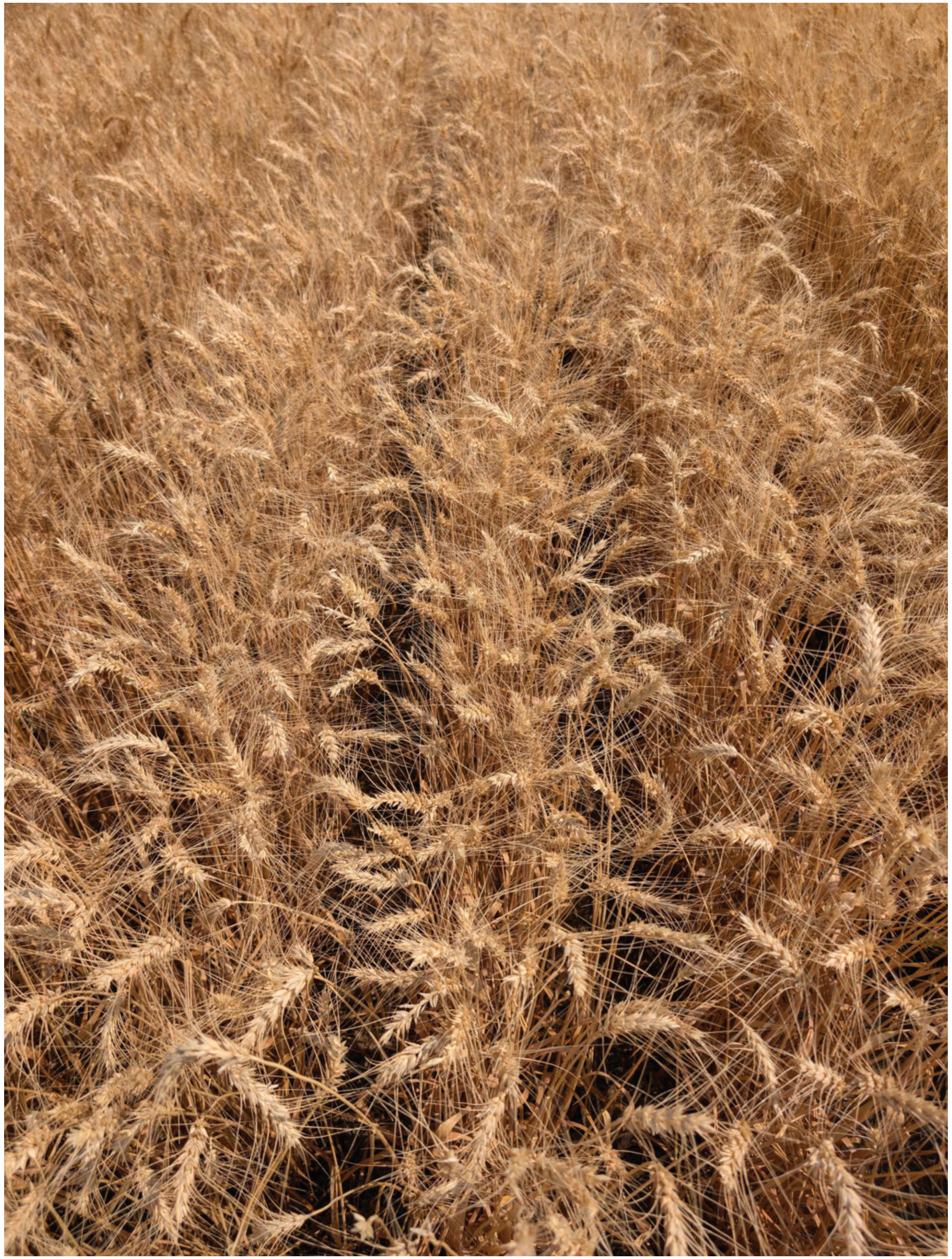

E

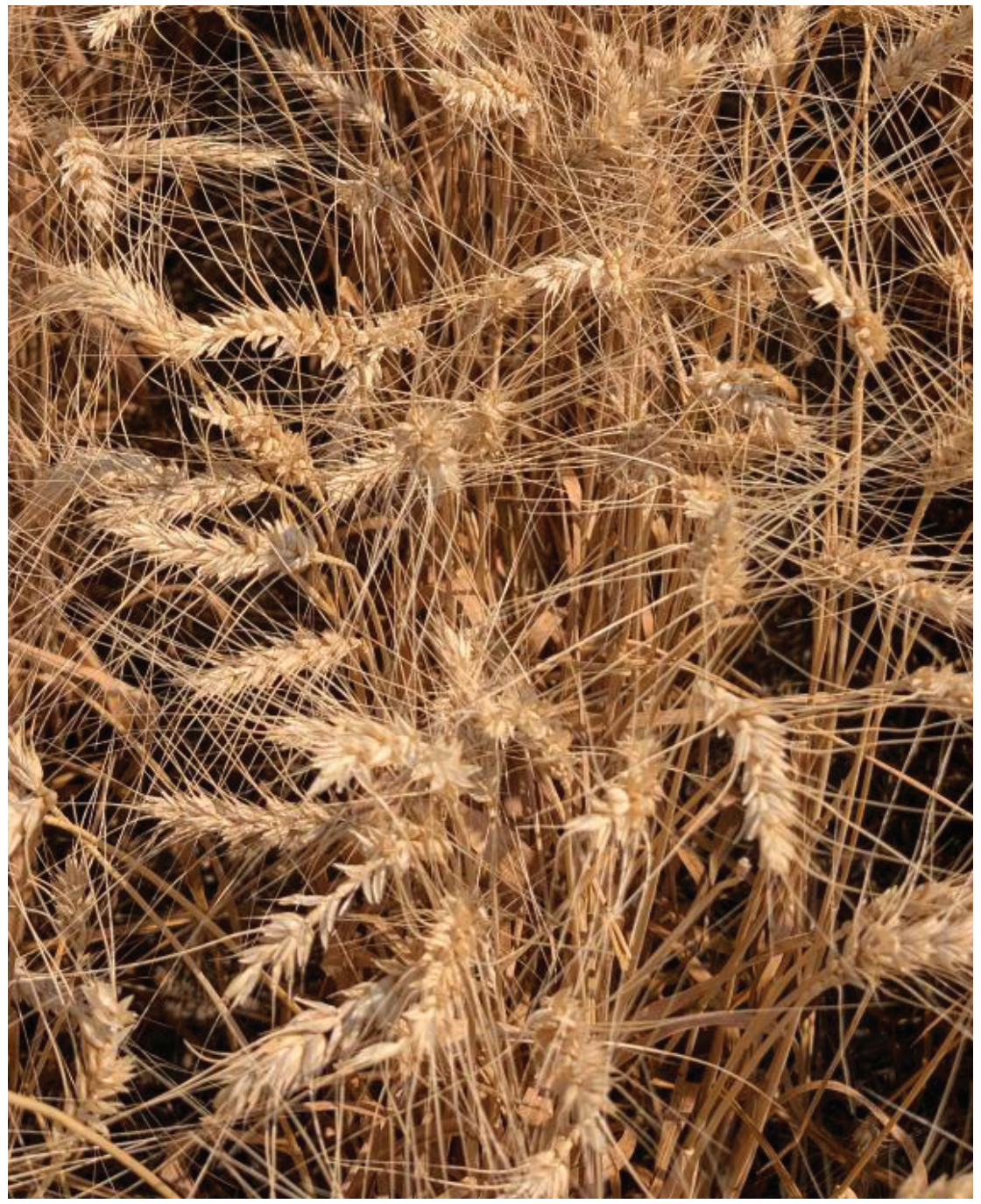

F

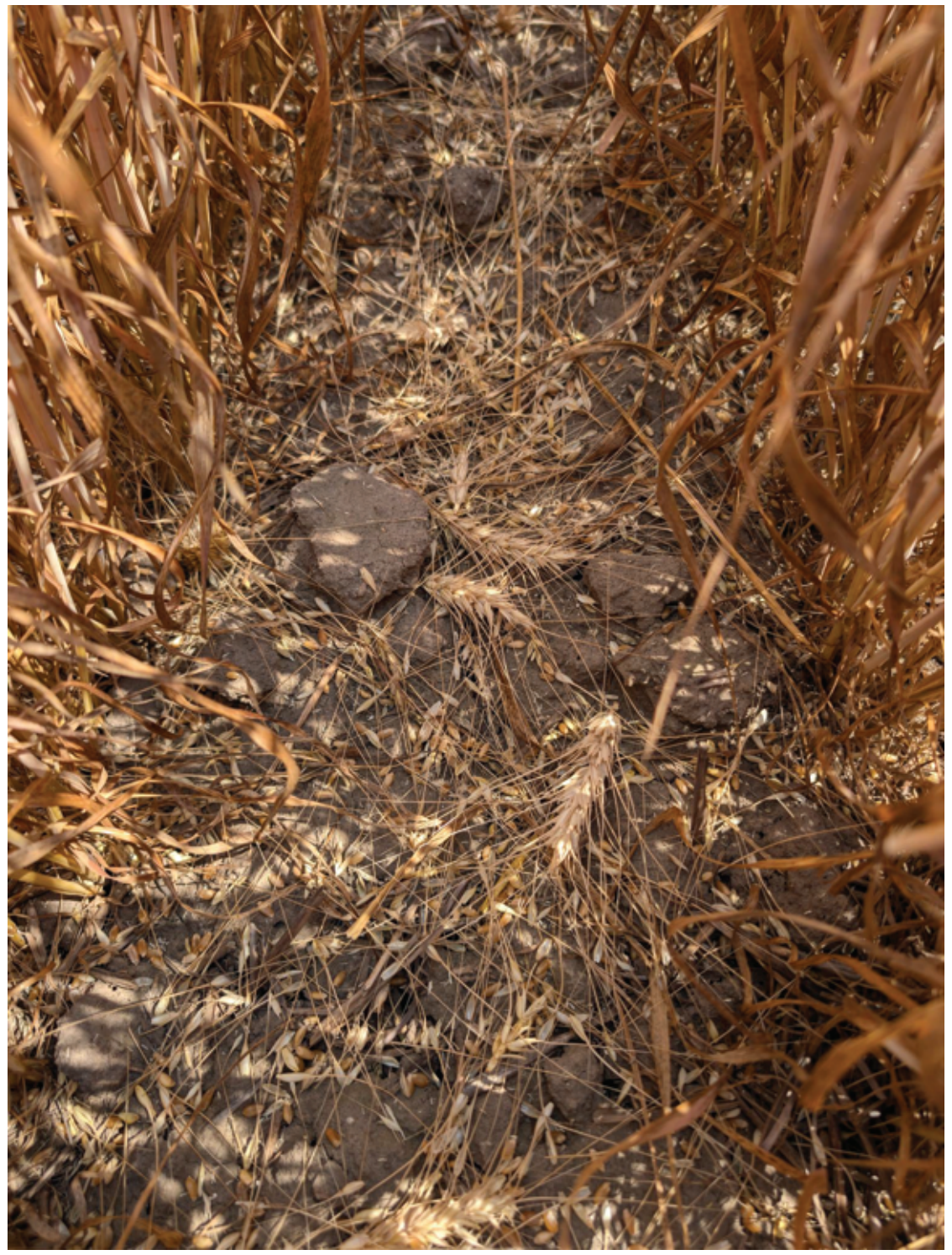

Figure S4. Effects of alternative alleles of singificant SNPs on grain yield (GY) and hail damage score (HLD)

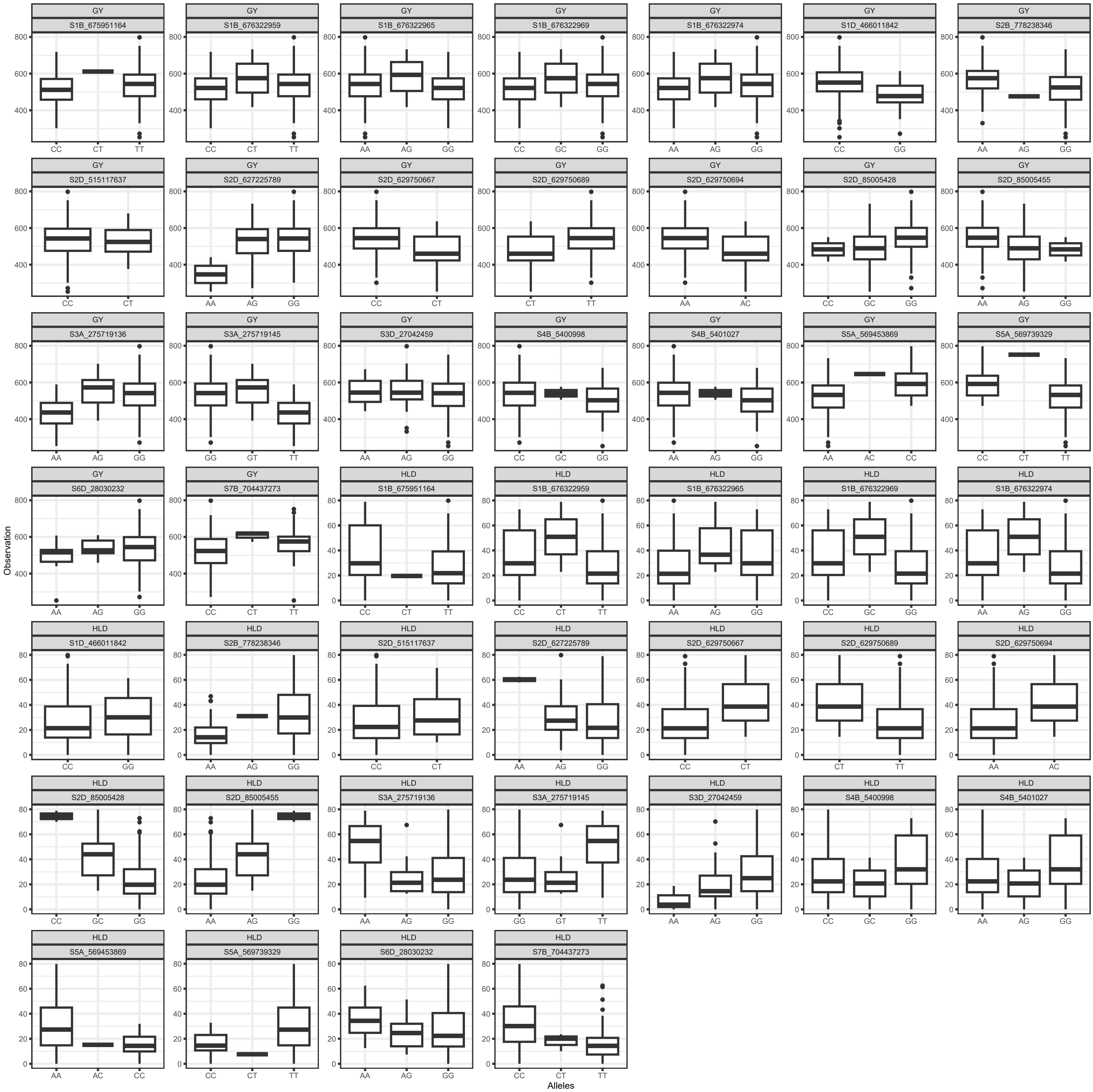

Supplement: Supplementary file 1 [file DataSheet1.pdf]
